# Cell-Based Bone Tissue Engineering

**DOI:** 10.1371/journal.pmed.0040009

**Published:** 2007-02-20

**Authors:** Gert J Meijer, Joost D de Bruijn, Ron Koole, Clemens A van Blitterswijk

## Abstract

The authors review the available data on bone tissue engineering and discuss possible new research areas that could help to make bone tissue engineering a clinical success.

To restore skeleton function in the field of orthopaedic and oral-maxillofacial surgery, bone tissue regeneration remains an important challenge. Spinal fusion, augmentation of fracture healing, and reconstruction of bone defects resulting from trauma, tumour, infections, biochemical disorders, or abnormal skeletal development are clinical situations in which surgical intervention is required. The types of graft materials available to treat such problems essentially include autologous bone (from the patient), allogeneic bone (from a donor), and demineralised bone matrices, as well as a wide range of synthetic biomaterials such as metals, ceramics, polymers, and composites.

Until recently, the use of autologous bone grafts has been the number one choice for bone repair and regeneration [1–5]. A patient's own bone lacks immunogenicity and provides bone-forming cells, which are directly delivered at the implant site. Moreover, autologous bone grafts recruit mesenchymal cells and induce them to differentiate into osteogenic cells through exposure to osteoinductive growth factors [1,3,6,7].

Although there are many advantages to using autologous bone, there are major drawbacks to the harvesting procedure, and for centuries there has been a search for alternatives. The extra surgery involved in harvesting autologous bone causes morbidity at the donor site [1,3,6,8] and can cause post-operative continuous pain [3,9–11], hypersensitivity [3], pelvic instability [10–12], infection [6,9], and paresthesia [3,6]. These complications affect 10% to 30% of the patients [9]. Moreover, the amount of bone that can be collected is limited.

As an alternative, the use of allografts (from human to human) eliminates the harvesting procedure and the quantity of available tissue is no longer an issue. Nevertheless, the quality of allografts is worse than that of autologous grafts. Allografts have a poor degree of cellularity, less revascularisation, and a higher resorption rate compared to autologous grafts [3,6], resulting in a slower rate of new bone tissue formation, as observed in several studies [11,13–15]. In addition, the immunogenic potential of these allografts and the risks of virus transmission to the recipient are serious disadvantages [2,14,16]. Although processing techniques such as demineralisation, freeze-drying, and irradiation have been shown to reduce the patient's immune response, processing also alters the structure of the graft and reduces its potential to induce bone healing (osteoinductivity), while the possibility of disease transmission still remains [3].

## Bone Tissue Engineering

To overcome the drawbacks of the current bone graft materials, bone tissue engineering (BTE) using bone marrow stem cells has been suggested as a promising technique for reconstructing bone defects. Indeed, various animal studies have shown the capacity of BTE to produce bone, both in a non-bone environment (ectopic bone formation) [17–27] and in a bone environment (orthotopic bone formation) [25,28–37].

**Five Key Papers on Bone Tissue Engineering**

Caplan,1991 [19] This author postulated that isolation, mitotic expansion, and site-directed delivery of autologous stem cells can govern the rapid and specific repair of skeletal tissues.

Friedenstein et al.,1987 [43] The authors showed that a specific set of cells (colony forming unit fibroblasts—CFU-F or MSC) existing in bone marrow can differentiate to different cell types, including osteoblasts.

Quarto et al., 2001 [52] The first clinical paper to report repair of large bone defects with the use of autologous bone marrow stromal cells.

Schimming et al., 2004 [53] The first study in humans showing that periosteum-derived osteoblasts can form lamellar bone within three months after transplantation.

Urist, 1965 [7] The author showed that bone tissue contains specific growth factors that can induce bone formation in ectopic sites.

Surprisingly however, until recently, no convincing successes have been achieved in humans. In this article, we review the available clinical data in the area of bone tissue engineering together with our own clinical experience. We discuss possible new directions that need to be exploited to make bone tissue engineering a clinical success.

## Search Strategy

We reviewed human studies published in international English language peer-reviewed literature regarding the treatment of osseous defects with tissue engineered constructs. We searched for studies in the Cochrane Central Register of Controlled Trials (Cochrane Library 2006, issue 2) and Medline (from 1966 up to March 2006). The search terms included culturing cells, mesenchymal, clinical, human study, orthotopic, ectopic, and bone formation. Publications presented in abstract form were ignored and case reports were excluded.

## Two Promising Approaches

To engineer the ideal bone graft material, factors that are capable of triggering osteogenesis must be included. Osteoinductive growth factors or progenitor cells [38] should be present or recruited. Apart from the use of gene therapy and embryonic stem cells, two novel bone engineering technologies promising to enhance bone healing have been introduced. Both techniques comprise three-dimensional scaffold structures that function as a carrier for growth factors or cells [3,6]. These technologies are therefore either growth factor–based or cell-based.

In the first approach, growth factors such as bone morphogenetic proteins of the TGF-â family are applied [6,39,40]. A potential drawback of this approach, however, is that high, supra-physiologic concentrations are needed to obtain the desired osteoinductive effect, with possible related side effects and high costs [41,42]. Furthermore, most if not all current techniques in which bone growth factors are used result in a burst-release of the growth factor shortly after placement followed by a limited release over longer time periods, thus in principle limiting the effectiveness of such an approach.

The second and more exciting approach is cell-based and combines living osteogenic cells with biomaterial scaffolds ex vivo to allow the development of a three-dimensional tissue structure. Below, we discuss this approach in more detail and focus on the first clinical results.

## The Cell-Based Approach

Since Friedenstein and colleagues' first publications in the 1980s [43], we have known that mesenchymal stem cells (MSCs) can be used to engineer mesenchymal tissues, such as bone and cartilage. Therefore, scientists worldwide are working to provide the right carrier and the appropriate set of cells that, once re-transplanted, will ensure bone repair.

Bone marrow has been claimed to be the most abundant source of MSCs, which have a high proliferative ability and great capacity for differentiation [17,44]. Also, from a practical point of view, bone marrow is an accessible source of osteogenic cells since it can be collected using a relatively simple aspiration procedure. As such, this method is less invasive than collecting osteogenic cells by taking biopsies from calvarium [45,46], periosteum [47], or trabecular bone [48]. Also, adipose-derived cells [49] and stem cells obtained from deciduous dental pulp [50] show osteogenic potential.

Caplan and colleagues combined MSCs with a scaffold to allow paracrine and host-derived factors to produce bone matrix after implantation. However, the proof of concept was mainly shown in ectopic rodent models [17–27], as well as in critical size defects in rodents [25, 28–31].

At this point, more than 300 papers about bone tissue engineering in rodents have been published indicating the feasibility of the technology. On the other hand, less than 10 studies have reported that orthotopic application (meaning in an osseous defect) is possible in, for example, segmental femur defects of larger animals such as dogs [32] or sheep [33,34]. Successful bone formation has also been reported in reconstructed skull [35] and mandibular defects [36] in sheep, and in iliac wing defects in goats [37]. Surprisingly, despite the promising future predicted by many of the above mentioned authors, only two studies have been published to date in humans, both claiming successful reconstructions [51–53].

### Clinical Studies.

The first clinical report described the treatment of three patients with various segmental defects (4 cm bone segment loss in the right tibia, 4 cm in the right ulna, and 7 cm in the right humerus), using ex vivo expanded human MSCs, loaded on a three-dimensional scaffold of the shape and size of the missing bone fragment [51,52]. External fixation was provided for stability and removed after 6.5, 6, and 13 months, respectively. All three patients presented a repair of the fracture site: the implants showed good integration of the newly formed bone and abundant callus formation.

However, conclusions were drawn based solely on radiographs; no biopsies were taken. Moreover, it would have been virtually impossible to observe bone formation in ceramic scaffolds on radiographs. Due to the high radiopacity of the ceramic material, the gain in radiopacity due to new bone formation is overshadowed by scattering. It is furthermore unclear if the callus formation is induced by the implanted human MSCs or by bone-forming cells in the periosteum.

The second published clinical study [53] describes the augmentation procedure of the posterior maxilla in 27 patients, using matrix derived from mandibular periosteum cells on a polymer fleece (Ethisorb; Ethicon, http://www.ethicon.com). In 12 patients, only radiographic and clinical assessments were performed. Limited conclusions can be drawn from the radiographic findings, as discussed above. The other 15 patients were treated according to a two-step method. First, reconstruction of the host area was performed. After a healing period of three months, in advance of dental implant placement, a biopsy was taken. In eight of these 15 patients an unsuccessful outcome was observed; a replacement resorption with connective tissue was found. In the case of a positive biopsy (seven patients), the authors failed to mention if the observed bone formation was possibly induced by the implanted cells (osteoinduction) or by the osteoblast from the pre-existing bone surface (osteoconduction).

In our own studies, we found that implanted cells were incapable of producing bone matrix in humans. In a pilot study (unpublished data), 10 patients with various intra-oral defects underwent reconstruction with cells cultured on a coralline hydroxyapatite (HA) scaffold. In only one patient did we find bone formation with histology strongly suggesting that the new bone was produced by the implanted cells (Figures 1 and 2). However, in a synchronously conducted control study, comparable cultured samples induced ectopic bone formation in seven out of 10 mice, underlining the weakness of the ectopic model in rodents to predict a successful clinical result.

**Figure 1:**
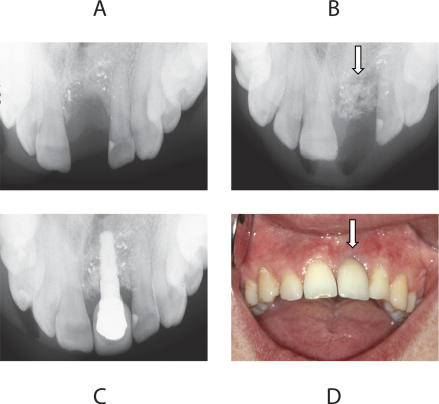
Alveolar Defect and Reconstruction with Tissue Engineering Patient (male, 20 years) has lost his upper left incisor during an accident. The alveolar defect was reconstructed with tissue engineering techniques. (A) Radiograph of the significant alveolar defect as a result of the accident. (B) Radiograph of the defect reconstructed with ceramic scaffold (HA) covered with cultured MSCs (white arrow). (C) Radiograph; four months later a dental implant was installed. (D) Intra-oral view; arrow points to the crown, which was fixated on the implant.

**Figure 2:**
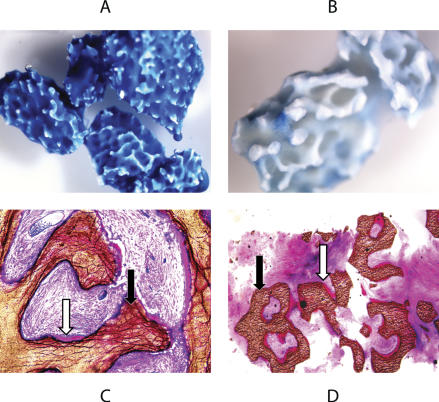
Samples and Histology of the Patient Shown in Figure 1 (A) HA particles stained with methylene blue immediately after seeding of the MSCs, showing cell distribution. (B) Idem stained with trypan blue after one week of culturing, showing cell vitality. (C) Histology six weeks after subcutaneous implantation in mice, showing in vivo bone formation (white arrow) in contact with HA particle (black arrow). (D) Histology after four months of implantation in the upper left tooth region, showing bone formation (white arrow) induced by the implanted cells in contact with the HA (black arrow).

## New Directions

From the above data it is apparent that clinical bone tissue engineering has not yet been a success. The crucial question therefore is: why do human MSCs fail to produce bone in an osseous defect, while the same cells do produce bone in an ectopic environment in mice?

For a successful outcome, four prerequisites are needed: (1) sufficient numbers of cells with osteogenic capacity; (2) an appropriate scaffold to seed the cells; (3) factors to stimulate osteogenic differentiation in vivo; and (4) sufficient vascular supply [19]. Evidently, all these four conditions are fulfilled ectopically and in critical size defects in rodents, because in these models MSCs seeded on a porous ceramic scaffold convincingly generated bone [18–23,25,28–31]. Up-scaling to humans appears to be highly challenging, as shown by the limited clinical reports [51–53].

The first three prerequisites can be fulfilled by engineering, while prerequisite number four is dependent on patient factors, such as the size of the defect. Lack of sufficient vascular supply, resulting in immediate cell death after implantation, is generally thought to be the cause of failure of BTE in patients [54].

The success of bone tissue engineering in ectopic rodent models is explained by the far more favourable biological environment for implanted cells. Often only a few small samples are subcutaneously implanted, which are in direct contact with the surrounding well-vascularised tissues. This shortens the diffusion depth, allowing the seeded MSCs to be optimally supplied by oxygen and nutrients. In addition, osseous defects in rodents are attractive sites for reconstruction. Defect sizes do not exceed the maximum distant depth of 5 mm, therefore allowing sufficient influx of oxygen and nutrition [55]. Moreover the remodelling speed in rodents is at least three times higher compared to humans [56].

New directions in research should therefore either be directed to the issue of solving the problem of inadequate diffusion and insufficient oxygen and nutrient supply for the cell-based approach, or to using biomaterial scaffolds that will recruit the appropriate osteogenic cells after implantation in the body. With regard to the former, only a sufficient number of new blood vessels within a short period of time guarantees an optimal survival rate of implanted cells. It has already been shown that improving vascularisation of tissue-engineered constructs can advance in vivo cell performance [57,58].

## Approaches to Improving Oxygen and Nutrient Supply

In the near future, several approaches to improve the oxygen and nutrient supply will be further investigated. One approach is to stimulate vessel growth by adding angiogenetic growth factors or endothelial cells to the tissue engineered construct. Especially in the case of the cell-based approach, vessel growth will be stimulated immediately after application [59].

A second method simply bypasses the problems linked to orthotopic bone formation by creating an engineered bone construct in a muscular environment (ectopic bone formation). Warnke et al. [60] reported a successful reconstruction of an extended mandibular discontinuity defect by growth of a custom bone transplant inside the latissimus dorsi muscle of an adult male patient. A prefabricated titanium mesh cage was filled with bone mineral blocks and infiltrated with 7 mg of recombinant human bone morphogenetic protein 7 and 20 ml of the patient's bone marrow. Thus prepared, the transplant was implanted into the latissimus dorsi muscle and seven weeks later transplanted as a pedicle bone-muscle flap to repair the mandibular defect. Although this experiment was considered successful, not only did the patient have to be operated upon twice and suffer from extra morbidity at the flap site, but no reliable assessment of bone formation was performed.

As a third approach, we suggest postponing the application of human MSCs for a few days after applying the scaffold. Immediately after implantation of the scaffold, a haematoma is formed [61,62]. On the third or fourth day, during the chronic inflammation phase, blood vessels and fibroblasts proliferate in the fibrin clot, thus forming granulation tissue [63]. By injecting the culture expanded MSCs at this time point, this approach ensures that the new blood vessels are already invading the haematoma, thereby guaranteeing a sufficient supply of oxygen and nutrients and thus securing the survival of the implanted cells. In addition, cells will be implanted at a time point during the wound healing process that the body would normally recruit stem cells to the defect site. Our recent unpublished data, comprising maxilla defects in goats, support this hypothesis. A comparative approach is advocated to regenerate heart tissue after infarction with the use of embryonic stem cell–derived cardiomyocytes [64].

An alternative direction for bone tissue engineering does not involve the pre- or peroperative use of stem cells and/or angiogenetic factors, but uses appropriate scaffolds that attract the patient's own stem cells post-implantation. This would circumvent all disadvantages of a cell therapy approach (MSC harvest and/or expansion prior to clinical use), and we have previously shown that such an in situ bone tissue engineering approach is feasible [65,66].

## Conclusions

Cell survival is the most important requirement for achieving clinical success in cell-based bone tissue engineering. Such cell survival can be promoted by various means such as: (1) co-culturing endothelial cells; or (2) bypassing the deleterious effect of the haematoma and lack of early vascularisation by a two-step implantation procedure: first the scaffold and approximately one week later injection of the MSCs. Another approach would be to ectopically implant the tissue engineering construct in a well-vascularised site in the body, i.e., muscle, to allow bone formation, followed by transplantation to the defect site. On-the-spot repair, which is currently obtained by autologous bone grafting, is still the optimal approach. What is indisputable is that MSCs are crucial for the healing of bone defects.

Besides the above mentioned cell-based techniques, another approach would be to recruit MSCs to the implantation site by growth factors or “smart” scaffolds. The use of these so-called osteoinductive scaffolds or one of the other mentioned alternative approaches could well revolutionise the future of regenerative medicine.

## Abbreviations

BTE: bone tissue engineering
HA: hydroxyapatite
MSC: mesenchymal stem cell
